# Hydrocarbon generation potential evaluation via petrographic and geochemical analyses of El-Maghara coal in Sinai, Egypt

**DOI:** 10.1038/s41598-024-51291-5

**Published:** 2024-01-09

**Authors:** S. A. Salman, E. A. Abou El-Anwar, W. A. Makled, K. H. Mahfouz, Z. L. Belal

**Affiliations:** 1 Geological Sciences Department, National Research Centre, 33 El Bohouth St. (Former El Tahrir St.), Dokki, POB 12622, Giza, Egypt; 2 Exploration Department, Egyptian Petroleum Research Institute (EPRI), 1 Ahmed El Zomor St. Nasr City, Cairo, 11727 Egypt; 3 Geology Department, Faculty of Science, Al-Azhar University, Assiut Branch, Asyut, 71524 Egypt

**Keywords:** Chemistry, Energy science and technology

## Abstract

The energy demand increased dramatically owing to the evolution of industrial and domestic requirements and the associated decrease in oil and gas resources. This study aims to evaluate El-Maghara coal (with about 52 MT reserve) as a potential hydrocarbon source. The collect samples were subjected to petrographic, chemical analyses and Rock–Eval pyrolysis to investigate the detailed characteristics of this coal. Chemically, this coal is high volatile bituminous coal with high H and S content. The high H/C ratio indicates the high extraction yield of coal. The main maceral group in the studied samples is vitrinite (62.8%) followed by liptinite (31.3%) and inertinite (5.8%). The content of liptinite indicates the capability of this coal for petroleum production. Based on Rock–Eval Pyrolysis results and TOC content, the coal has excellent petroleum potential. The hydrogen index (HI) and H/C atomic ratio indicate the II kerogen type (oil prone) of this coal. This coal has T_max_ and vitrinite reflectance values around 415.8 °C and 0.37%, respectively, indicating the immature stage of kerogen. The high reactive maceral content (94.2%), oil-yield (65.5%) and conversion from coal to oil (95.4%), indicated that this coal has a hydrocarbon generation potential for oil.

## Introduction

The world overpopulation and associated urbanization, industrial and technological revolution increased the demand for energy. Coal is one of the first energy resources that is mined. The future is for coal as an energy source because oil and gas reserves are diminishing very fast, while coal reserves are plentiful^1^. Based on the IEA^2^, coal will continue to be the main energy source for the steel industry in 2050. So, the understanding of coal characteristics and improvement of the comprehensive parameters of coal for metallurgical applications is an important issue^3^.

Coal occupies the second place worldwide as an energy source after oil and natural gas^4^. The rapid growth of technology and population in the last decades has led to an increasing demand for energy sources (liquid and gaseous hydrocarbons) in most countries of the world^5^. To fill the shortfall in petroleum and natural gas, start searching for unconventional sources of hydrocarbons such as Coalbed methane, oil shale, tar sands and gas hydrate^6–8^. It shows that many countries, such as the USA, Indonesia, China and Australia, have managed to generate hydrocarbons from these sources^9–11^.

El-Maghara coal seams in Sinai follow the Jurassic age and consists of about 11 lenticular seams, including a main economic layer with a thickness ranging from 1.3 to 2 m. Coal ash minerals consist mainly of quartz with some calcite, anhydrite and hematite. Maghara coal can be classified as medium volatile bituminous coal. Coal with high sulfur content, especially pyritic sulfur, indicate a possible marine intrusion after the deposition of the peat precursor. The bituminous rank of El-Maghara coal with its volatile matter content (> 37.8%) supports the generation of methane from this coal^7^. Low rank coal (lignite) have exploited for hydrocarbon potential in India^12–16^. Also, sub-bituminous per-hydrous coals with high H/C ratio of Indonesia possess high conversion and oil yield^17^. The chemical study showed that El-Maghara coal is distinguished from the worldwide coal by its low concentrations of environmentally harmful trace and rare elements^18^. El-Maghara coal petrographic study revealed the dominance of vitrinite, followed by liptinite and inertinite, while the mineral matter includes mainly clay, quartz, and pyrite^19,20^. This organic and mineral composition indicated the predominance of anoxic waterlogged conditions in the mire during peat formation^19^.

El-Maghara Activated coal shows effective removal of Pb and Zn in alkaline medium^21^. El-Maghara coal was used to produce Nano-activated carbon (alkaline and thermal activation), which showed effective results as an adsorbent for removing methyl orange dye from industrial wastewater^22^.

Coal is mainly a source of gas, but it can also be a source of oil under some circumstances^23^. The importance of coal as a source of petroleum has increased dramatically in recent decades, and many studies worldwide have pointed out many new approaches regarding the oil and gas formation of humic coal (e.g. Refs.^24–29^). Karayigit et al.^30^ pointed out the importance of using complete physical and chemical analyses of low rank to avoid misinterpretations of source rock. In the current work, petrographic, proximate, ultimate and Rock–Eval pyrolysis analyses were applied to interpret the possibility of hydrocarbon generation from El-Maghara coal.

Previous studies have focused on the general characteristics (petrography, calorific value, proximate and ultimate data), reserves, geological settings and depositional environment of El-Maghara coal (e.g. Refs.^19,31–33^). This study, mainly, aims to identify the properties and hydrocarbon generation potential of El-Maghara Jurassic coal, North Sinai, Egypt. It also, aims to provide an organic geochemical assessment of these coal seams using proximate and ultimate analyses, petrographic, total organic carbon (TOC), vitrinite reflectance (R_o_) and Rock–Eval pyrolysis data.

## Materials and methods

### The study area

**Figure 1:**
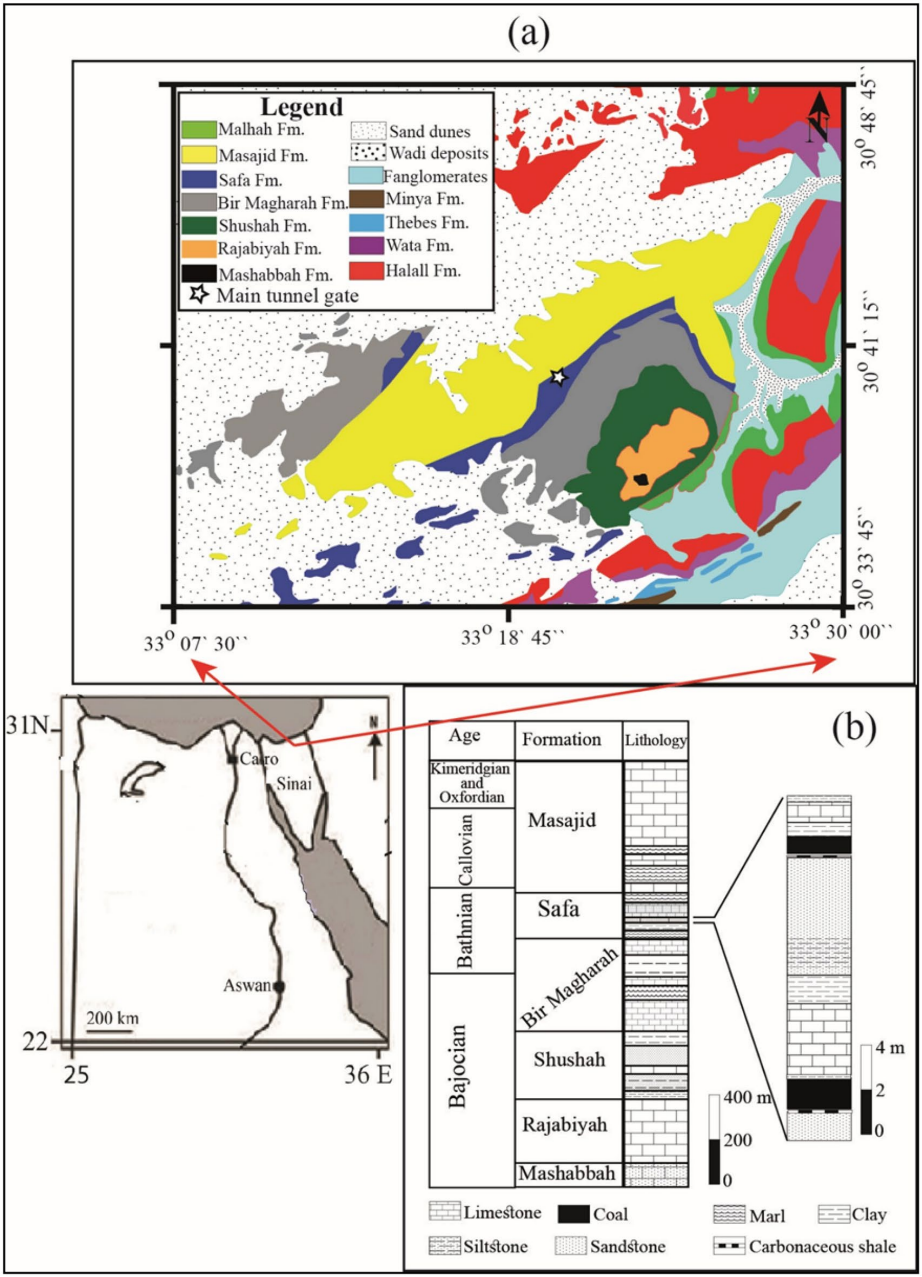
(**a**) Geological map of El-Maghara area (modified after EGSMA^36^), (**b**) Stratigraphic column of exposed formations at El-Maghara^31^.

El-Maghara area is located approximately in the center of North Sinai, about 200 km northeast of Cairo, between longitudes 33°10' and 33°40' E, and latitudes 30°35' and 30°50' N. It is a rectangular sedimentary basin with an area of about 1300 km^2^ (Fig. 1a). The region occupies great scientific and economic importance because it contains the ideal Jurassic section in Egypt as well as economic coal deposits. Therefore, it has been subjected to many geological studies (e.g. Refs.^31,32,34,35^). Al-Far^31^ divided the Jurassic sediments in the region into six formations (Fig. 1b) and described them in detail. Safa Formation has particular importance because it contains the economic coal layer, the geological reserve of coal is estimated at 52 million tons^32^.

El-Maghara coal mine has faced many problems since its establishment in 1964. The mine was closed due to the 1967 war for 15 years, which led to the destruction and collapse of the mine facilities. In 1982, mine rehabilitation operations began with the help of the British company, Babcock, but the mine was closed again in 2005 due to some technical and financial problems. One of the reasons that led to the closure of the mine is that coal can’t be coked except by mixing it with a higher grade of coal, and that Egypt does not contain coal-fired power stations^37^. However, due to the problem of energy shortage facing many countries in the world, the use of El-Maghara coal to generate energy or liquefying it to obtain oil and gas using modern technologies should be reconsidered.

### Physico-chemical analyses

The proximate and ultimate analyses were performed according to ASTM procedures in the Egyptian Mineral Resources Authority; Moisture^38^, volatile matter^39^, ash^40^, calorific value^41^, total sulfur^42^, CHN^43^, fixed carbon and oxygen were calculated by difference. The concentrations of measured proximate and ultimate analyses were calculated based on instructions in ASTM^44^ and Suggate^45^.

Rock–Eval pyrolysis was used to measure free hydrocarbons (S1 = mg HC/g rock), residual hydrocarbon generating potential (S2 = mg HC/g rock), free CO_2_ (S3 = mg HC/g rock) and temperature during maximum generation of hydrocarbons at S2 (T_max_ °C) of the coal sample. LECO SC632 was used for TOC at Stratochem service lab, Egypt. These parameters were used to calculate the hydrogen index (HI), oxygen index (OI), potential yield (PY) and production index (PI)^46^.

$${\text{HI}} = { 1}00 \times {\text{ S2}}/{\text{TOC}}$$

$${\text{OI }} = { 1}00 \, \times {\text{ S3}}/{\text{TOC}}$$

$${\text{PY }} = {\text{ S1 }} + {\text{ S2}}$$

$${\text{PI}} = {\text{S1}}/\left( {{\text{S1}}/{\text{S2}}} \right).$$

The figures were processed with Adobe Illustrator CS5 software for re-drawing and enhancement^47^. In the present study, organic petrography is used to explore the maceral content and thermal maturity in 6 samples that were collected from the coal seam (Table 1). The analysis is conducted in the whole rock samples that were consolidated in epoxy resin and polished according to the procedures indicated in the ASTM^48^. The maceral composition is quantified by calculating the area percentages of each type from the photomicrographs by Image J software in incident light including white and fluorescence modes. The thermal maturity is measured using the procedures of ASTM^49^ to calculate the vitrinite reflectance (R_o_ %). The maceral content, especially reactive macerals (RM), can be used to determine oil yield and conversion of coal into hydrocarbon^50,51^ using the following formulas^52^.

$${\text{Conversion }}\left( \% \right) \, = \, 0.{\text{2RM }} + { 76}.{6}$$

$${\text{Oil }} - {\text{ yield }}\left( \% \right) \, = \, 0.{\text{22RM }} + { 44}.{8}$$

$${\text{RM}} = {\text{ Vitrinite}}\% \, + {\text{ Liptinite}}\% .$$

**Table 1:** Coal measured and calculated data of petrography, proximate, ultimate and Rock–Eval.

Parameter	1	2	3	4	5	6	Mean	Min	Max
Proximate									
Moisture %	1.71	2.94	2.39	2.74	2.87	2.25	2.48	1.71	2.94
VM %	43.16	42.34	44.48	46.96	45.1	44.03	44.35	42.34	46.96
Ash %	11.1	9.82	3.18	4.29	10.03	16.97	9.23	3.18	16.97
Fixed carbon %	44.03	44.9	49.95	46.01	42	36.75	43.94	36.75	49.95
Ultimate									
C %	71.31	64.36	77.35	73.61	63.03	63.26	68.82	63.03	77.35
H %	7.43	6.63	7.65	7.87	6.2	6.25	7.01	6.2	7.87
N %	1.1	0.96	1.08	1.12	0.85	0.91	1	0.85	1.12
S %	3.32	7.64	2.97	3.81	7.23	4.57	4.92	2.97	7.64
O %	4.1	7.82	5.51	6.75	10.08	5.92	6.7	4.1	10.08
Calorific value MJ/kg	34.35	30.59	36.43	35.33	29.08	29.73	32.59	29.08	36.43
FR	1.02	1.06	1.12	0.98	0.93	0.84	0.99	0.83	1.12
H/C	1.25	1.24	1.19	1.28	1.18	1.19	1.22	1.18	1.28
O/C	0.04	0.09	0.05	0.07	0.12	0.07	0.07	0.04	0.12
C/N	75.61	78.19	83.53	76.65	86.48	81.08	80.26	75.61	86.48
Rock–Eval data									
S1 (mg HC/g rock)	6.87	6.80	7.62	7.83	5.16	7.30	6.93	5.16	7.83
S2 (mg HC/g rock)	233.7	215	240.7	265.4	206.7	231.1	232.1	206.7	265.4
S3 (mg HC/g rock)	10.33	10.54	9.03	10.79	10.03	8.85	9.93	8.85	10.79
T_max_ (°C)	419	412	414	416	413	421	415.8	412	421
TOC (wt%)	66	62.6	63.9	67.8	58.9	58.1	62.9	58.1	67.8
HI (mg HC/g TOC)	354.1	343.4	376.6	391.4	350.9	397.8	369.1	343.4	397.8
OI	15.65	16.84	14.13	15.91	17.03	15.23	15.80	14.13	17.03
PI	0.03	0.03	0.03	0.03	0.02	0.03	0.03	0.02	0.03
PY	240.6	221.8	248.3	273.2	211.9	238.4	239	211.9	273.2
S2/S3	22.62	20.39	26.65	24.59	20.61	26.12	23.50	20.39	26.65
Vitrinite%									
Telovitrinite	3	2	3	3	6	4	3.5	2.0	6.0
Detrovitrinite	52	59	57	54	60	9	48.5	9.0	60.0
Gelovitrinite	2	1	2	2	15	43	10.8	1.0	43.0
Sum	57	62	62	59	81	56	62.8	56.0	81.0
Liptinite %									
Cutinite	12	10	13	9	3	7	9.0	3.0	13.0
Suberinite	3	1	1	3	1	3	2.0	1.0	3.0
Sporinite	5	3	6	4	1	5	4.0	1.0	6.0
Resinite	5	7	3	5	1	4	4.2	1.0	7.0
Exsudatinite	0	4	0.5	1	1	5	1.9	0.0	5.0
Alginite	2	4	0	0	0	2	1.3	0.0	4.0
Liptodetrinite	9	6	12	13	2	2	7.3	2.0	13.0
Bituminite	2	0	0.5	0	0	7	1.6	0.0	7.0
Sum	38	35	36	35	9	35	31.3	9.0	38.0
Inertinite%									
Fusinite	1	0	1	1	9	2	2.3	0.0	9.0
Funginite	2	1	0.5	1	1	2	1.3	0.5	2.0
Secretinite	1	3	0.5	4	2	3	2.3	0.5	4.0
Sum	4	4	2	6	12	7	5.8	2.0	12.0
RM%	95	97	98	94	90	91	94.2	90.0	98.0
Conversion%	95.6	96	96.2	95.4	94.6	94.8	95.4	94.6	96.2
Oil – yield%	65.7	66.14	66.36	65.48	64.6	64.82	65.5	64.6	66.36

## Results and discussion

### Proximate and ultimate analyses

The proximate analysis result of El-Maghara coal (Table 1); indicated low moisture content (2.48%, as received) and medium ash yields (9.23%, as received), high calorific values (32.59 MJ/kg, on dry, ash-free basis). The coal samples display high volatile mater (44.35%, as received) and fixed carbon (43.94%, as received) values. This moderate ash content may have resulted from the process of mixing mineral materials with organic materials during the sedimentation process due to geological factors such as the rate of sedimentation, changing water levels, and tectonic processes^53^. Edress et al.^19^ pointed out the sea level fluctuations and associated water table changes during coal deposition in El-Maghara. Also, the microscopic composition of the studied coal assesses in the preservation of mineral mater because vitrain is characterized by cleats as well as micro-fractures and pores, which can trap mineral matter^54^.

**Figure 2:**
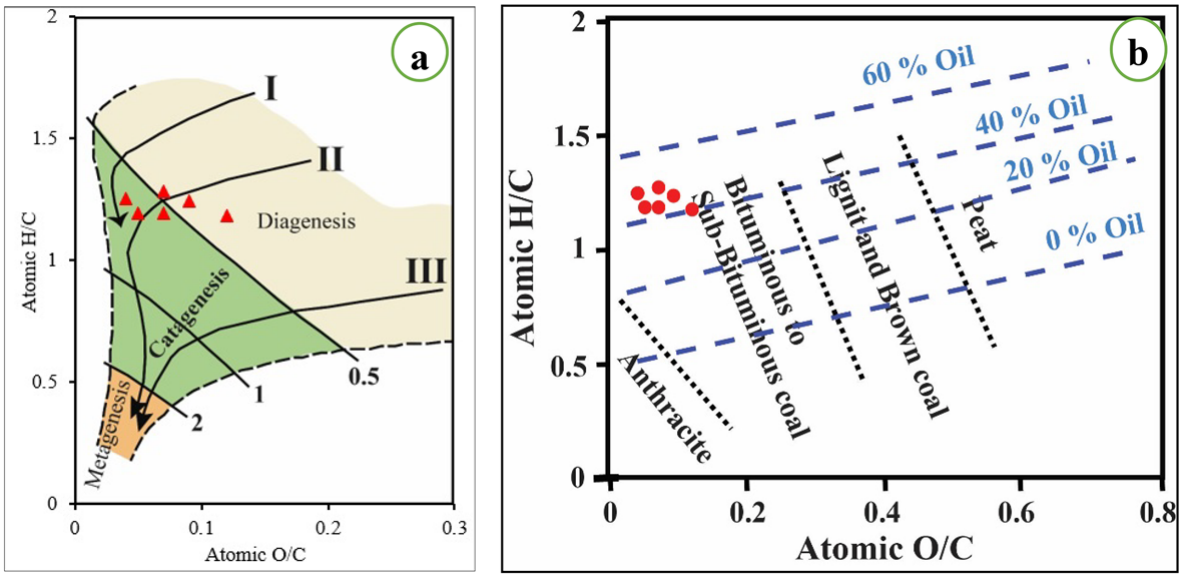
(**a**) Van Krevelen diagram showing data for the samples from El-Maghara, (**b**) Plot of H/C versus O/C for rank determination (after^58^), blue dashed lines for oil yield after Saxby^59^.

The results of the ultimate analysis, on a dry, ash-free basis, of El-Maghara coal are listed in Table (1). The studied coal samples have high C (68.82%), H (7.01%), total S (4.92%) and N (1%). The high S content of this coal is related to its depositional environment, where this coal was deposited in marine anoxic conditions^19^. This is confirmed by the recorded sulfur minerals; pyrite and copiapite^33^. The coal content of H controls the physical properties of OM; the transform of OM from solid to liquid to gas increases with increasing H content^23^. The H content has a positive correlation with Rock–Eval S1 (r = 0.68) and S2 (r = 0.83). The atomic ratio of H/C was about 1.22 and O/C was 0.07 in the El-Maghara coal (Table 1). This relatively high H/C ratio may be resulted from the abundance of H-rich macerals; derovitrinite and liptinite. The high H/C ratio is good indicative of the high extraction yield of coal^55^. These atomic ratios, H/C and O/C, were used by Van Krevelen^56^ to determine kerogen type and hydrocarbon generation potential. The studied coal samples were plotted between Types I and II kerogens on the Van Krevelen diagram (Fig. 2a), indicating the richness of these samples with H (perhydrous coal). The H/C atomic ratio can be used for kerogen type discrimination; H/C > 1.4 indicates type I (oil-prone), 1 < H/C < 1.4 indicates type II (oil-prone), 0.4 < H/C < 1 indicates type III (natural gas-prone) and H/C < 0.4 indicates type IV (barren)^57^. Accordingly, the studied coal samples, with H/C = 1.22, are of type II kerogen and have the potential for petroleum production. Also, the atomic H/C—O/C plot (Fig. 2b)^58^ can be applied to determine the studied coal rank; the studied coal samples have bituminous rank.

### Petrography

**Figure 3:**
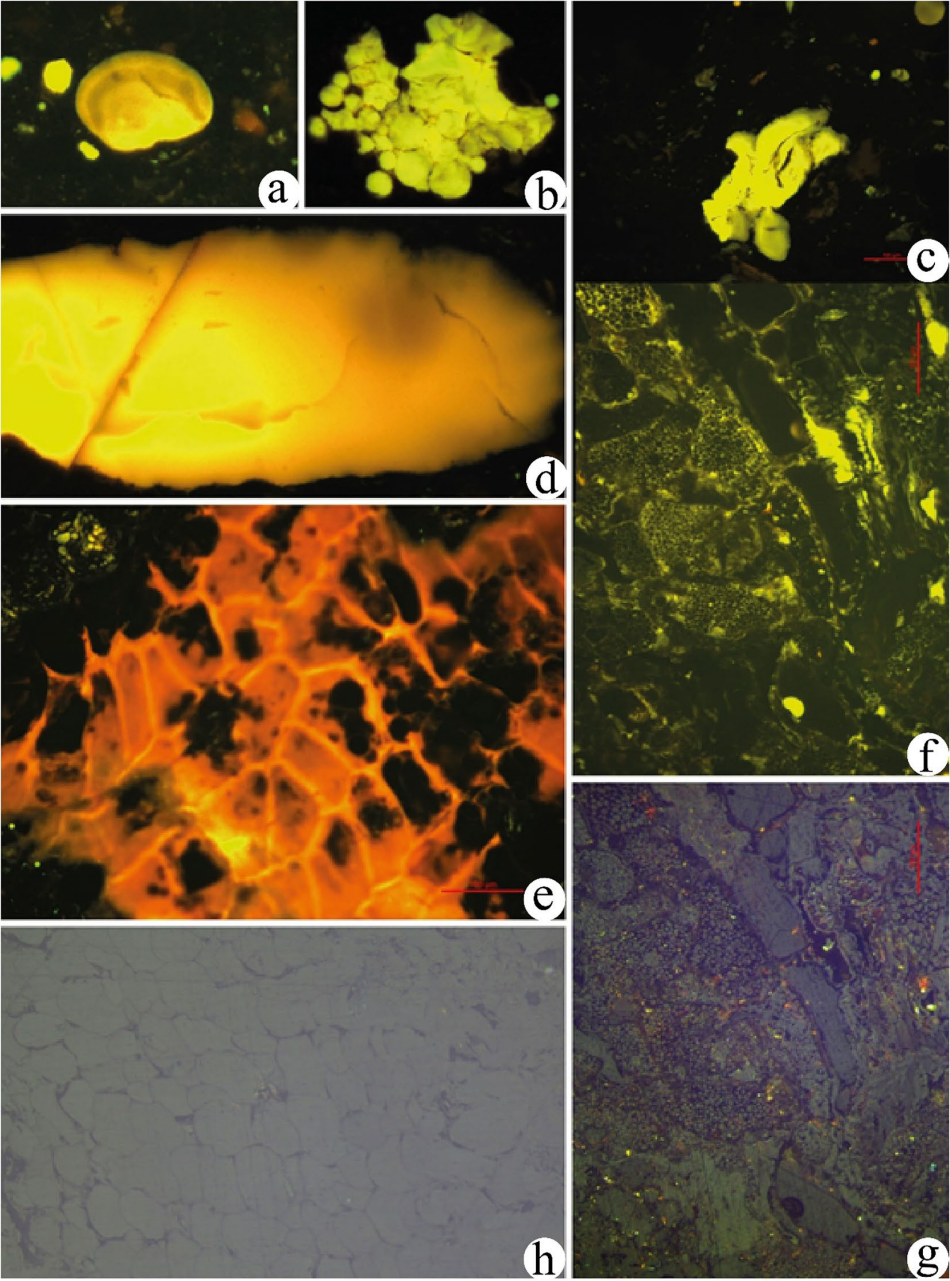
(**a**) Sporinite in fluorescence light, sample 1 (**b**) Exsudatinite in fluorescence light, sample 2 (**c**,**d**) Resinite in fluorescence light, sample 3 (**e**–**g**) Cutinite of different origin and structure in Vitrinite matrix, (**e**) and (**f**) in fluorescence light, g in white light, sample 3 (**h**) Vitrinite in cell structure in whit light, sample 3.

The maceral composition is distinguished by diversity of components that comprise vitrinite, liptinite and inertinite (based on dry mineral matter free). The dominant maceral is vitrinite including detervitrinite (9–60%), gelovitrinite (2–43%) and telovitrinite (3–10%) (Table 1; Fig. 3f–h). Liptinite is exclusively of terrigenous composition including sporinite (1–5%), cutinite (1–13%), suberinite (1–5%), resinite (1–7%), exsudatinite (0–6%), alginite (0–6%), liptodetrinite (2–13%) and bituminite (0–14%) (Table 1; Fig. 3a–g)). The liptinite macerals are characterized by bright strong fluorescence colors that ranges from yellow to light orange (Fig. 3). Inertinite occurs in minors, including fusinite (0–14%), funginite (1–15%) and secretinite (0–4%) (Table 1; Fig. 3g). The vitrinite reflectance is measured in a sample (5C) and (3C). The mean value is 0.37, which indicates thermally immature coal. This in accordance with the light and bright fluorescence colors of liptinite.

**Figure 4:**
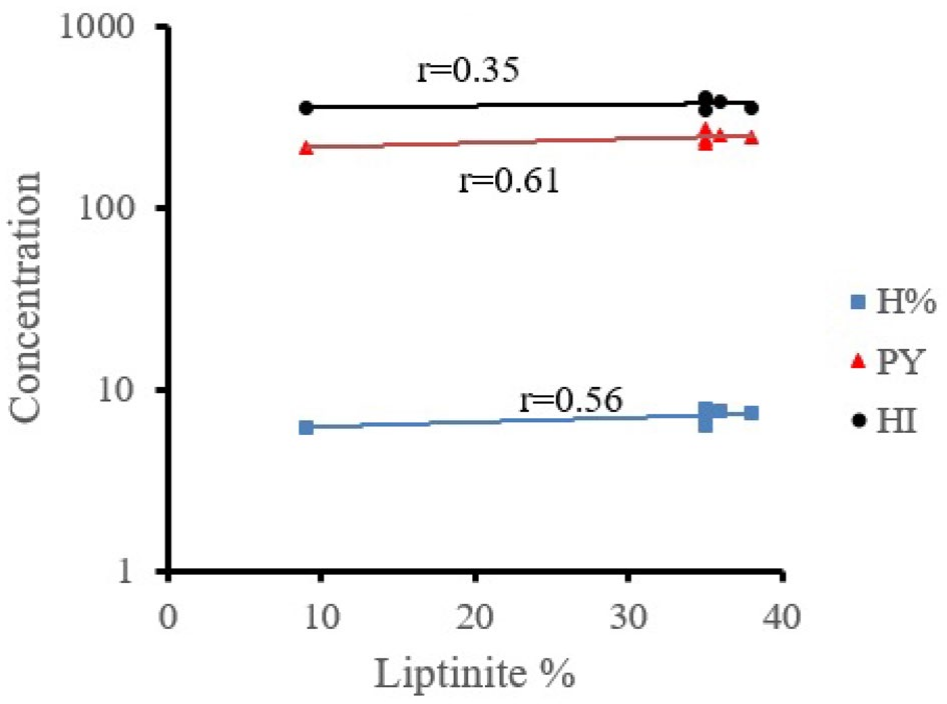
Relationship between liptinite and H%, Py and HI.

The coal content of liptinite is in forward proportion with the H%, HI and PY (Fig. 4), because liptinite macerals are H-rich and contain more aliphatic compounds than vitrinite and hence more oil-prone^60^. The presence of more than 12% liptinite is responsible for the recorded high HI (> 350 mg HC/g TOC) (Table 1)^61^.

### Coal rank

**Figure 5:**
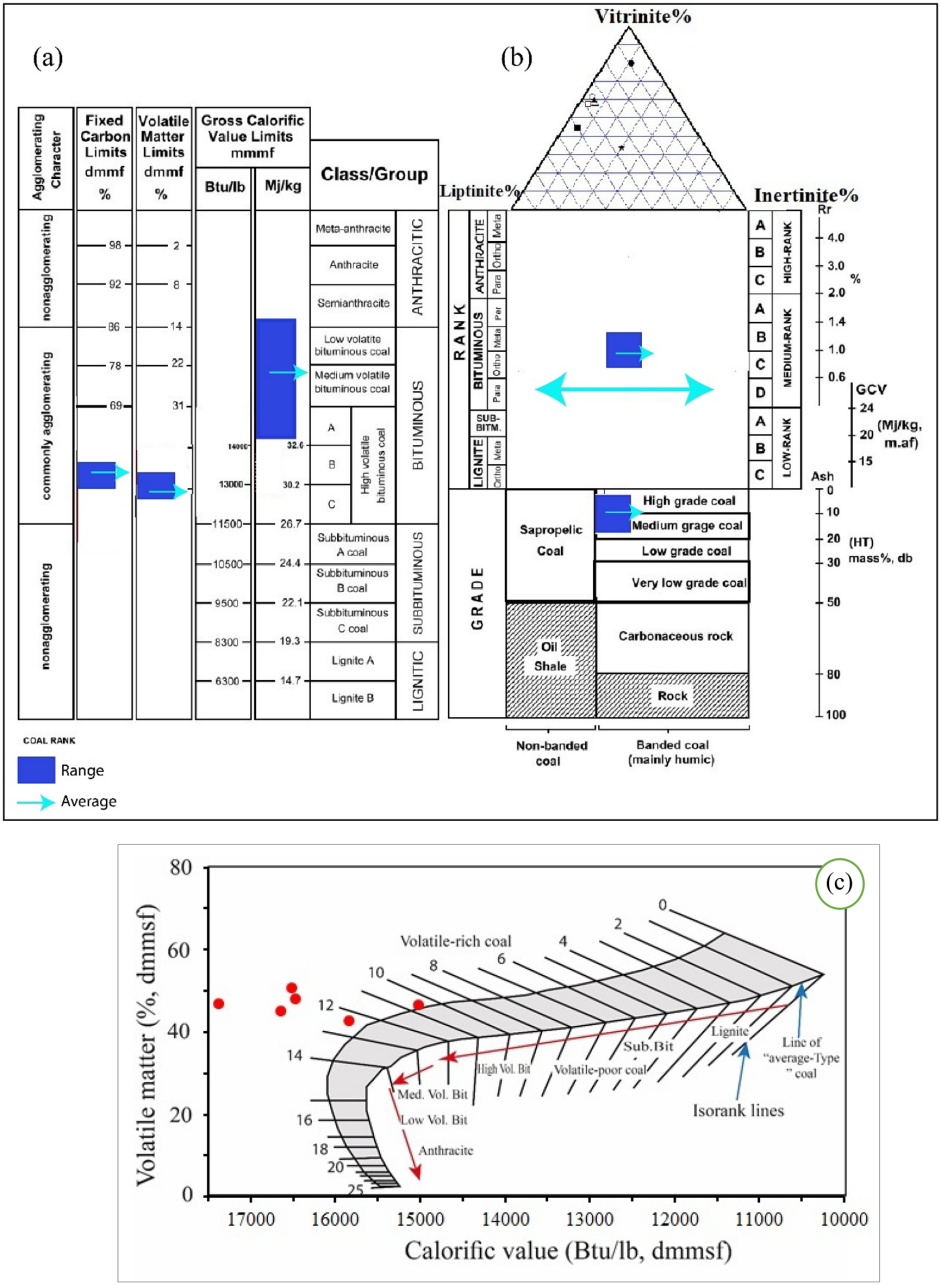
Coal rank classification (**a**) based on Ref.^44^, (**b**) based on Ref.^63^, (**c**) Coal rank based on CV and VM (After Ref.^45^).

Coal rank refers to the coal organic matter metamorphism (coalification), or coal maturity and measure of the degree of coal evolution from peat to meta-anthracite^62^. High rank coal has high R_o_, C and C/H ratio, and low VM, and vice versa^3^. So, coal rank can’t be determined through a single parameter but by using many physical and chemical parameters; R_o_, moisture, calorific value, volatile matter and fixed carbon. The plotting of these parameters on the ECE-UN^63^ and ASTM^44^ systems (Fig. 5a,b) indicated medium to high volatile bituminous coal^44^ which is correspondence to medium rank bituminous coal^63^. This is confirmed by the plot of calorific value-volatile matter (Fig. 5c) of Suggate^45^. Accordingly, this coal is suitable for producing coal bed methane^53,64–66^. The fuel ratio (FR) of the studied coals was around 0.99, placing the samples at the bituminous rank based on Frazer’s^67^ classification. These results are in agreement with Edress and Abdel-Fatah^68^.

### Organic geochemistry

**Figure 6:**
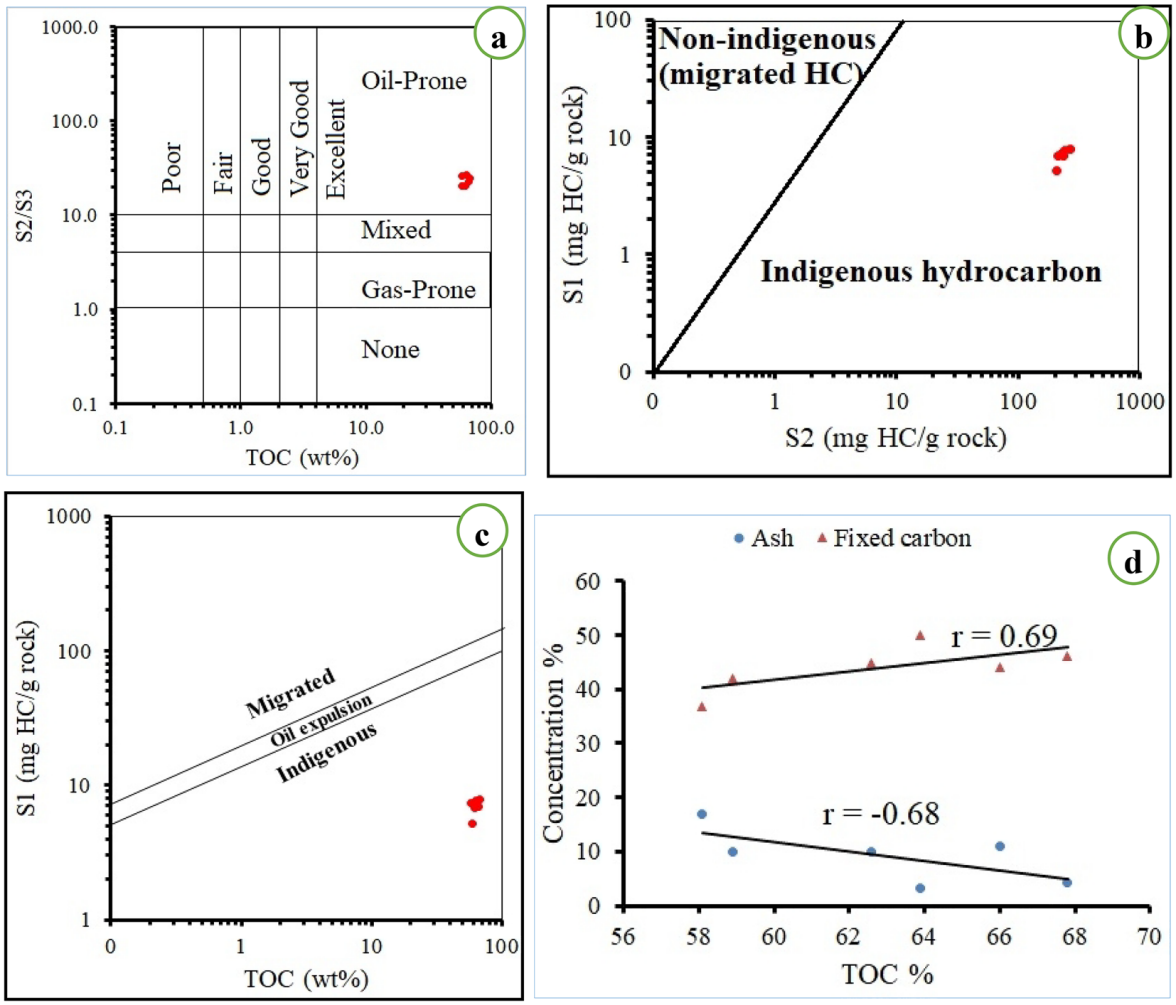
(**a**) Plots of TOC versus S2/S3 ratio, (**b**) Plots of S1 versus S2, (**c**) Plots of TOC versus S1, (**d**) plot of TOC vs fixed carbon% and ash%.

Table (1) illustrates the results of Rock Eval pyrolysis (S1, S2, S3 and Tmax), TOC, and R_o_, which can be used to characterize coal organic richness and ability to generate hydrocarbons^69,70^. The values of these parameters were 5.2–7.8 mg HC/g rock, 206.7–265.4 mg HC/g rock, 8.9–10.8 mg HC/g rock, 412–421** °C**, 58.1–67.8 wt%, 0.4; respectively. Based on the S1 (> 4), S2 (> 20) and TOC (> 4) the studied samples are of excellent petroleum generative potential (Table 1). The hydrocarbon richness value (S2/S3) of El-Maghara coal samples ranged between 20.39 to 26.65, which indicates the definite possibility of generating oil from those samples (Fig. 6a). The results of the study (S1, S2 and TOC values) showed that the organic matter in coal is indigenous hydrocarbons, not migratory ones (Fig. 6b,c).

The positive correlation of TOC (r = 0.69) with the fixed carbon (Fig. 6d) infers that both of them play an essential role in determining how well coal can be used as a potential source rock. As known, high rank coal has high fixed carbon and somewhat high TOC. On the contrary, the inverse correlation between TOC (r = − 0.68) and ash (Fig. 6d), indicates that the increase of mineral matter during the coal deposition process implicitly decreases the availability of organic carbon and thus negatively affects the ability of coal to produce hydrocarbons.

#### Thermal maturity

**Figure 7:**
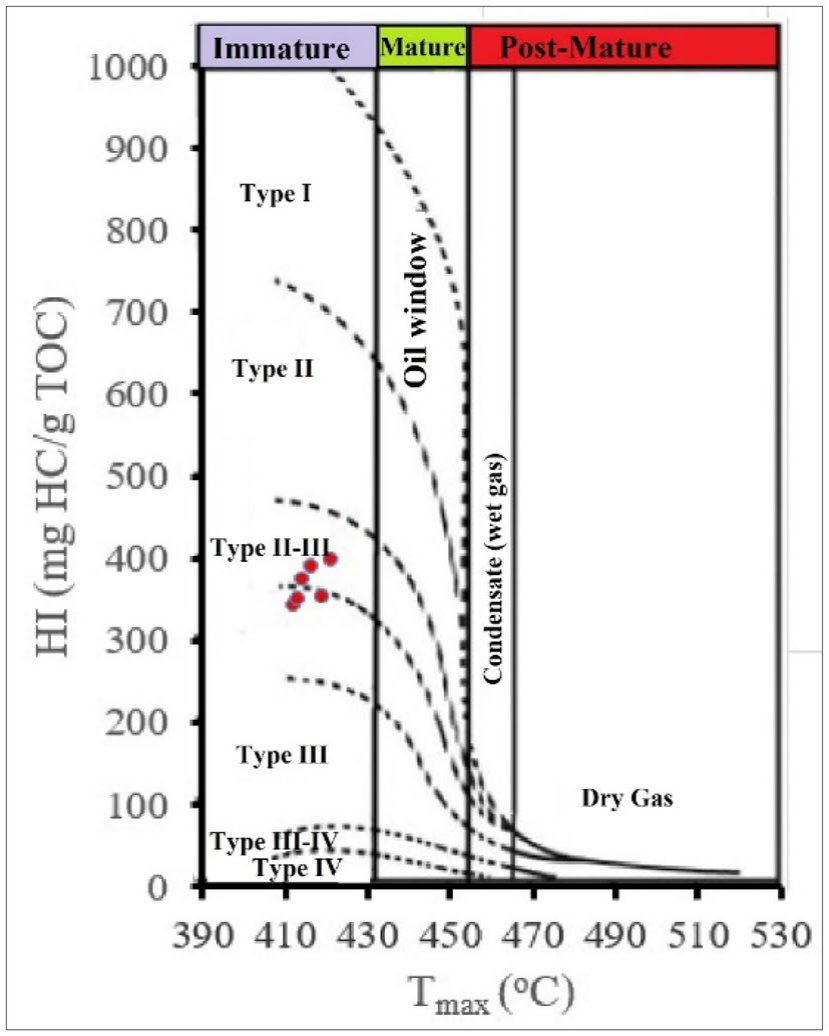
Pseudo-Van Krevelen diagrams of Hydrogen Index (HI) versus T_max_.

The thermal maturity of kerogen plays an essential role in determining the ability of source rocks to generate hydrocarbons as well as the types of hydrocarbons (gas or oil). The thermal maturity of source rocks is determined by several maturity indicators, such as T_max_ values, production index (PI), and vitrinite reflectance (R_o_)^71–73^. The T_max_ values in El-Maghara coal samples ranged from 412 to 421 °C, which indicates the immaturity of kerogen, as identified from Fig. 7. This is confirmed by the low values of R_o_, which were less than 0.6, as well as the low production index values, which ranged between 0.02 and 0.03.

#### Hydrocarbon generation potentiality

The hydrocarbon generation ability of kerogen is assessed in this study from the petrographic composition, kerogen type, thermal maturity, genetic potential, HI values, and TOC content. The oil generation potential mainly depends on the liptinite content in the source rock; petroleum production requires > 15% liptinite^23,25,74^. The studied coal samples have liptinite content of around 31.3%, indicating the capability of this coal for petroleum production. Coal liquefaction potentiality is controlled by its content of reactive macerals (vitrinite and liptinite)^14,75^. Coal with Ro < 0.8, reactive macerals > 60% and volatile component (daf) > 35% is suitable for liquefaction and gasification^76,77^. The studied samples composed mainly of reactive macerals, varying from 90 to 98 wt % (Table 1), indicating their suitability for hydrocarbon generation. In addition, the calculated conversion (94.6–96.2%) to oil and oil yield (64.6–66.4%) (Table 1), illustrates the liquid hydrocarbon generation potential of this coal^78^.

**Figure 8:**
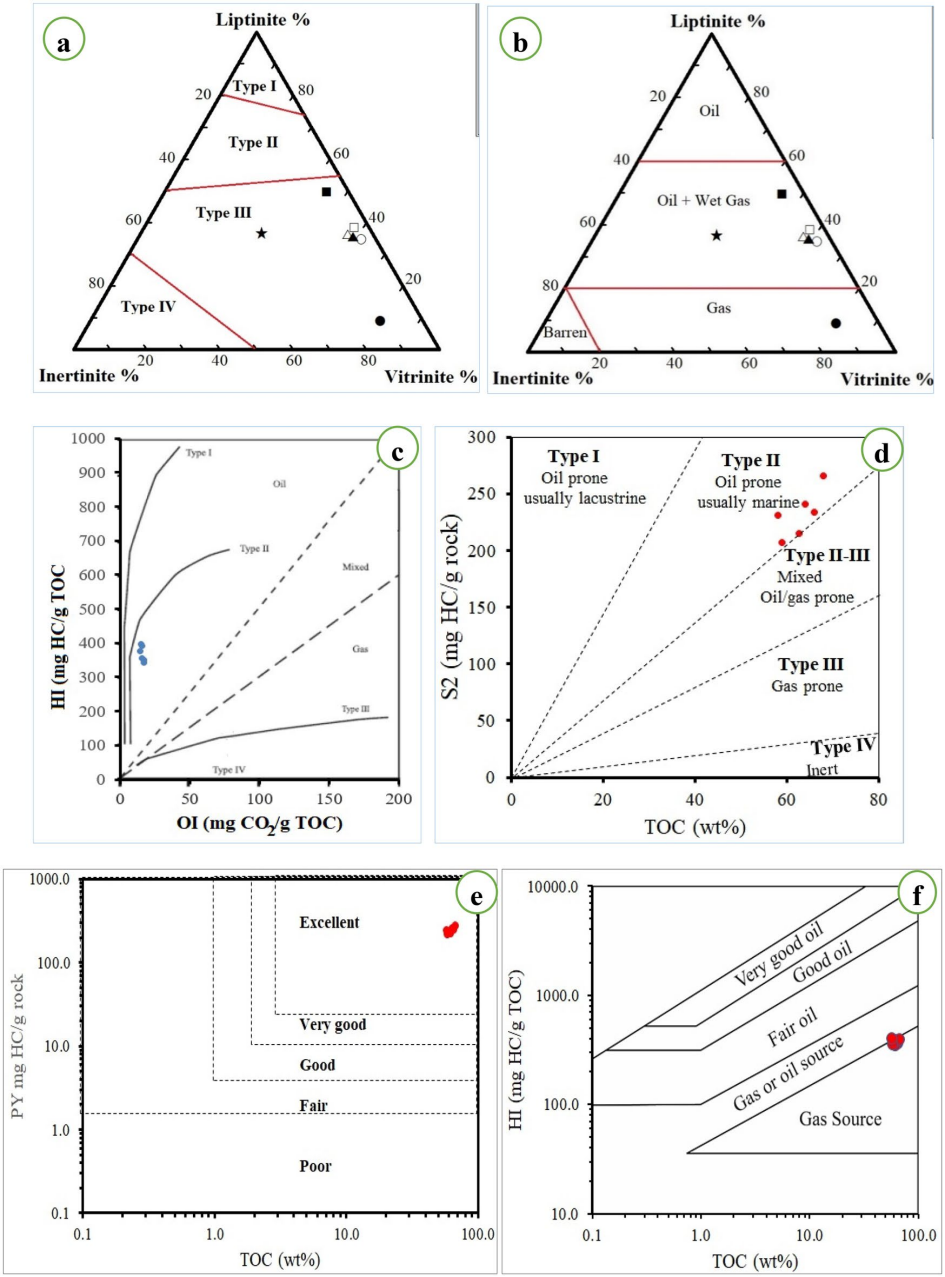
Ternary diagrams of maceral composition indicating (**a**) kerogen types (After Ref.^50^), (**b**) hydrocarbon type (after Ref.^79^), (**c**) plot of OI vs HI, (**d**) plot of TOC vs S2, (**e**) Plot of TOC vz Py, (**f**) relationship between TOC and HI.

The ternary plot of maceral composition was applied to deduce the kerogen and hydrocarbon type. The samples are mainly of Vitrinite (62.8%) followed by liptinite (31.3%), so the samples contain type III kerogen (Fig. 8a) and have excellent probability for hydrocarbon generation; oil and gas (Fig. 8b).

The hydrocarbon potential of coal depends mainly on the amount and type of organic matter and its thermal maturity^80,81^. The type of kerogen depends on the source of the organic matter, which largely controls the possibility of generating hydrocarbons and its type; Type I and II come from algae and are considered oil-prone, while type III comes from higher plants and is considered gas-prone^57^. Since the organic matter is mainly composed of C, H and O, the kerogen type in coal can be determined using the hydrogen index (HI)^82^.

El-Maghara coal recorded values ranging from 343.39 to 397.83 mg.HC/g.TOC For HI, which shows that the kerogen in those samples is type II, as illustrated in Fig. 8c. This is confirmed by the S2-TOC (Fig. 8d), which indicates that these samples can produce oil. The high content of coal from hydrogen-rich macerals (detrovitrinite and liptinite) also indicated the ability of this coal to produce oil^83^.

The PY values of El-Maghara coal samples were 211.9–273.2 mg HC/g rock, indicating their excellent hydrocarbon generation efficiency based on Hunt’s scale^84^, which is confirmed by the PY-TOC diagram (Fig. 8e). The relationship HI-TOC (Fig. 8f) also indicated that El-Maghara coal samples represent a source of gaseous hydrocarbons in addition to oil. The high H content of the studied coal (H > 5), H/C ratio > 0.9 and liptinite content > 15%, indicate its great capability to generate oil and gas^23^. The current results support the importance of using petrographic, proximate and ultimate analyses, inside Rock–Eval pyrolysis results, as concluded by Karayigit et al.^30^, for a more accurate interpretation of source rock hydrocarbon generation.

### Comparison of the current results with others

The results of the studied coal samples were compared to the previous studies on El-Maghara coal (Table 2), and agreement was observed between the current and previous results, which indicates the consistency of the coal layer in the mine and the accuracy of the studies and analyses. It was noted that previous studies didn’t use the Rock–Eval pyrolysis technique to determine the amount of organic matter and the extent of the possibility of producing hydrocarbons from this coal.

**Table 2:** Comparison of the current results with previous studies from Egypt and worldwide.

Proximate and ultimate analysis	Current study	M1	M2	M3	Turkey^30^	India^51^	China^85^	Colombia^86^
Moisture % (a.r)	1.71–2.94 (2.48)	5.5	3.03	1.97	–	6	–	–
Ash % (d.b)	3.3–17.4 (9.5)	14.1	7.54	7.14	43.7	8.3	7.1	5
Volatile matter % (d.b)	43.6–48.3 (45.5)	45.4	52.64	52.22	35.8	42.5	55.71	44.6
Fixed carbon % (d.b)	37.6–51.2 (45.1)	40.5	39.82	40.6	20.5	49.1	44.29	55.4
Carbon % (d.a.f)	72.1–81.8 (77.7)	77.3	76.3	71.2	80.7	65.33	81.8	–
Hydrogen % (d.a.f)	7.1–8.5 (7.9)	6.72	6.2	6.0	5.5	5.42	6.22	–
Nitrogen % (d.a.f)	1–1.3 (1.1)	1.48	1.3	1.2	2.4	2.53	–	–
Sulfur % (d.a.f)	3.1–8.7 (5.6)	1.2o	4.3	4.1	3.5	2.32	–	0.6
Oxygen % (d.a.f)	4.7–11.5 (7.6)	13.3	11.9	17.6	7.8	24.4	–	–
Gross Calorific Value (MJ/kg)	29.08–36.43 (32.59)	–	30.33	30.04	20.3	–	–	–
vitrinite reflectance %	0.37	0.44		0.43		0.4	–	–
TOC	58.1–67.8 (62.88)	–	–	–	15.7–51.5	65.55	69.54	75.6
S1	5.16–7.83 (6.93)	–	–	–	0.3–6.4	7.51	8.17	2.2
S2	206.7–265.37 (232.09)	–	–	–	5.3–83	173.94	240.65	191.9
S3	8.85–10.79 (9.93)	–	–	–	14.1–53.6	9.04		4.8
T_max_	412–421 (415.83)	–	–	–	389–436	419	439	426.7
HI	343.39–397.83 (369.05)	–	–	–	32–161	271	346.1	254.4
OI	14.13–17.03 (15.8)	–	–	–	71–123	14		6.4
PI	0.02–0.03 (0.03)	–	–	–	0.02–0.07 (0.03)	0.04	0.03	0.01

M1: El-Maghara coal (after Ref.^87^).

M2: El-Maghara coal (after Ref.^18^).

M3: El-Maghara coal (after Ref.^68^).

El-Maghara coal was also compared with coal from Turkey, India, China and Columbia. It was observed that El-Maghara coal is nearly identical with the Indian coal. It has more volatile matter than Turkey, India and Columbia as well as less TOC than India, China and Columbia and has PI equal to Turkey and China, less than India, and more than Columbia.

## Conclusion

El-Maghara coal mine is located in North Sinai and contains a geologic reserve of 52 million tons. The mine was closed due to the poor quality of coal and its high sulfur content. The results of the current study indicate that the coal contains a high amount of volatile matter and is of the bituminous type and consists mainly of vitrinite macerals. The elevated hydrogen content in this coal indicates its perhydrous nature. The high H/C ratio, attributed to the abundance of H-rich macerals (derovitrinite and liptinite), is a good indication of the high extraction yield of this coal. The reactive macerals content represents about 94.2% of the studied coal samples, of them 31.3% liptinite indicating the suitability of El-Magara coal for liquefaction and gasification. Generally, the high H content of the studied coal (H > 5), H/C ratio > 0.9 and liptinite content > 15%, indicate its great capability to generate oil and gas. The amounts of organic matter in this coal are high (TOC = 62.9% and S2 = 232.1 mg HC/g rock), but immature (T_max_ = 415.8 °C). The S1, S2 and TOC values indicate the indigenous organic matter in coal. The hydrocarbon richness value (S2/S3) of El-Maghara coal samples points out the possibility of generating oil from those samples. However, the current study showed that El-Maghara coal has the potential to generate hydrocarbons; oil and gas. The current study also clarified the importance of combining petrographical, chemical and physical studies of low-grade coal to obtain more accurate results about the properties, rank and coal ability to produce energy.

## Data Availability

All the data are provided within the manuscript.

## References

[1] Towler BF, Towler BF (2014). Coal and clean coal technologies. The Future of Energy.

[2] IEA (International Energy Agency) https://www.iea.org/energy-system/industry/steel#overview.

[3] Lu L, Osborne D (2023). Utilization parameters of coal for metallurgical applications. The Coal Handbook.

[4] Caldwell, R.L. Energy Outlook. Forage and Grain: A College of Agriculture Report, University of Arizona (Tucson, AZ). https://repository.arizona.edu/bitstream/handle/10150/199709/370051-078-084.pdf?sequence=1 (2023).

[5] BP. Statistical Review of World Energy 2023. BP, London. (2023).

[6] Mastalerz M, Drobniak A, Mastalerz M, Drobniak A (2020). Coalbed methane: Reserves, production, and future outlook. Future Energy.

[7] Ojha S, Punjrath NK, Chakraborty A, Singh PK (2023). Evolution and evaluation of coal-bed methane in Cambay Basin, Western India: Insights from stable isotopic and molecular composition. J. Geol. Soc. India.

[8] Shah S, Totlani K (2014). Difficulties and prospects of Coalbed methane in India as compared to North America. J. Unconvent. Oil Gas Resour..

[9] Islam MR (2014). Unconventional Gas Reservoirs: Evaluation, Appraisal, and Development.

[10] Ma YZ, Holditch S (2015). Unconventional Oil and Gas Resources Handbook: Evaluation and Development.

[11] Thakur P (2020). Coal Bed Methane: Theory and Applications.

[12] Singh PK (2012). Petrological and Geochemical considerations to predict oil potential of Rajpardi and Vastan lignite deposits of Gujarat, Western India. J. Geol. Soc. India.

[13] Singh PK (2016). Eocene lignites from Cambay basin, Western India: An excellent source of Hydrocarbon. Geosci. Front..

[14] Singh PK, Singh VK, Rajak PK, Mathur N (2017). A study on assessment of hydrocarbon potential of the lignite deposits of Saurashtra Basin, Gujarat (Western India). Int. Jour. Coal Sci. Tech..

[15] Singh VP (2017). Investigation on the lignite deposits of Surkha mine (Saurashtra Basin, Gujarat), western India: Their depositional history and hydrocarbon generation potential. Int. J. Coal Geol..

[16] Rajak PK (2021). Study of hydrocarbon source potential of Kapurdi lignites of Barmer basin, Rajasthan, western India. J. Geol. Soc. India.

[17] Singh PK, Singh MP, Singh AK, Arora M, Naik AS (2013). The prediction of the liquefaction behavior of the East Kalimantan Coals of Indonesia: An appraisal through petrography of selected coal samples. Energy Sour. A.

[18] Baioumy HM (2009). Mineralogical and geochemical characterization of the Jurassic coal from Egypt. J. Afr. Earth Sci..

[19] Edress NA, Oplustil S, Sýkorova I (2018). Depositional environments of the Jurassic Maghara main coal seam in north central Sinai, Egypt. J. Afr. Earth Sci..

[20] Melegy AA, Salman SA (2009). Petrological and environmental geochemical studies on the abandoned Maghara coal mine. Geolines.

[21] Melegy A (2010). Adsorption of Lead (II) and Zinc (II) from Aqueous Solution by Bituminous coal. Geotech Geol Eng.

[22] Elkady M, Shokry H, Hamad H (2020). New activated carbon from mine coal for adsorption of dye in simulated water or multiple heavy metals in real wastewater. Materials.

[23] Hunt JM (1991). Generation of gas and oil from coal and other terrestrial organic matter. Org. Geochem..

[24] Petersen HI (2002). A re-consideration of the "oil window" for humic coal and kerogen type III source rocks. J. Pet. Geol..

[25] Petersen HI (2006). The petroleum generation potential and effective oil window of humic coals related to coal composition and age. Int. J. Coal Geol..

[26] Kotarba MJ, Lewan MD (2004). Characterizing thermogenic coalbed gas from polish coals of different ranks by hydrous pyrolysis. Org. Geochem..

[27] Zeng L (2020). Assessment of oil potentials for humic coals on the basis of flash Py-GC, Rock-Eval and confined pyrolysis experiments. Org. Geochem..

[28] Quan Y (2022). Hydrocarbon generation potential, geochemical characteristics, and accumulation contribution of coal-bearing source rocks in the Xihu Sag, East China Sea Shelf Basin. Mar Pet Geol.

[29] Petersen HI, Fyhn MBW, Nytoft HP, Dybkjær K, Nielsen LH (2022). Miocene coals in the Hanoi Trough, onshore northern Vietnam: Depositional environment, vegetation, maturity, and source rock quality. Int. J. Coal Geol..

[30] Karayigit AI, Oskay RG, Çelik Y (2021). Mineralogy, petrography, and Rock-Eval pyrolysis of late Oligocene coal seams in the Malkara coal field from the Thrace Basin (NW Turkey). Int. J. Coal Geol..

[31] Al-Far DM (1966). Geology and coal deposits of G. Maghara, N. Sinai. General Egyptian Organization for geological research and mining geol. Surv. Pap..

[32] Mostafa AR, Younes MA (2001). Significance of organic matter in recording paleoenvironmental conditions of the Safa Formation coal sequence, Magara Area, North Sinai, Egypt. Int. J. Coal Geol..

[33] Salman SA (2008). Study of Some Environmental Impacts on Maghara Coal Mine Area.

[34] Jenkins D.A. North and central Sinai. In: R. Said (Eds), *The Geology of Egypt*, 361– 380 (1990).

[35] Issawi B, El Hinnawi M, Francis M, Mazhar A (1999). The phanerozoic geology of Egypt: A geodynamic approach. Geol. Surv. Egypt.

[36] EGSMA. Geological map of Al Maghara quadrangle, Sinai, Egypt, scale 1:100000, Sheet No. NH 36 K4. Ministry of Petroleum and Mineral Resources, Geological Survey of Egypt. (1993).

[37] Wafiq A, Hanafy M (2015). Feasibility assessment of diesel fuel production in Egypt using coal and biomass: Integrated novel methodology. Energy.

[38] ASTM D3173. Test Method for Moisture in the Analysis Sample of Coal and Coke: Gaseous Fuels; Coal and Coke, 5. International. Pennsylvania, ASTM, p. 6 (2005).

[39] ASTM D3175, 2004. Standard method of volatile matter in the analysis sample of coal and coke from coal. In: Annual Book of ASTM Standards 2004. Gaseous Fuels: Coal and Coke, vol. 05.06. ASTM, Philadelphia, PA, pp. 327–330.

[40] ASTM D3174, 2004. Standard method of ash in the analysis sample of coal and coke from coal. In: Annual Book of ASTM Standards 2004. Gaseous Fuels: Coal and Coke, vol. 05.06. ASTM, Philadelphia, PA, pp. 322–326.

[41] ASTM D5865, 2004. Standard test method for gross calorific value of coal and coke. In: Annual Book of ASTM Standards 2004. Gaseous Fuels: Coal and Coke, vol. 05.06. ASTM, Philadelphia, PA, pp. 519–529.

[42] ASTM D3177, 2002. Standard Test Methods for Total Sulfur in the Analysis Sample of Coal and Coke (withdrawn 2012). ASTM International, West Conshohoken, PA, USA.

[43] ASTM D5373, 2004. Standard test methods for instrumental determination of carbon, hydrogen, and nitrogen in laboratory samples of coal and coke. In: Annual Book of ASTM Standards, Part 26. Gaseous Fuels: Coal and Coke ASTM, Philadelphia, PA, pp. 504–507.

[44] ASTM (American Society for Testing and Materials) D388–99. Standard Classification of Coals by Rank. ASTM International, West Conshohocken, PA (7 pp) (2000).

[45] Suggate RP (2000). The Rank (Sr) scale: Its basis and its applicability as a maturity index for all coals. New Zealand J. Geol. Geophys..

[46] Espitalie, J., Madec, M., Tissot, B., Mennig, J.J. & Leplat, P., Source rock characterization method for petroleum exploration. In: *Proceedings of the 9*^*th*^* Annual Offshore Technology Conference*, pp. 439–444 (Paper OTC 2935). (1977).

[47] Adobe illustrator software. https://www.adobe.com/products/illustrator.html.

[48] ASTM, D 2797–4. Standard Practice for Preparing Coal Samples for Microscopical Analysis by Reflected Light (5 pp.). ASTM International, West Conshohocken, PA (2004).

[49] ASTM, D7708–14. Standard Test Method for Microscopical Determination of the Reflectance of Vitrinite Dispersed in Sedimentary Rocks. ASTM, International, West Conshohocken, PA, USA, pp. 10 (2014).

[50] Nath M (2023). New insight into geochemical characterization of Paleogene coals from Jarain coalfield, Meghalaya, N-E India: Hydrocarbon potential and organic petrographic analysis. Geoenergy Sci. Eng..

[51] Gogoi M, Kumar TS, Sarat PS (2020). Organic geochemistry, petrography, depositional environment and hydrocarbon potential of the eocene coal deposits of west Daranggiri coalfield, Meghalaya. J. Geol. Soc. India.

[52] Jin, J. & Shi, S. The Development and Prospective Application of Coal Direct Liquefaction for Chinese Coals. Proceeding of international symposium on clean coal technology, Xiamen, China Coal Industry Publishing House, 379 (1997).

[53] Kumar S, Varma AK, Mendhe VA, Kumar S, Bhan U (2022). Geochemical and petrographical fingerprints of coal bed methane potential in the Son-valley Basin. India. Arabian Journal of Geosciences.

[54] Rai S (2022). Study of micro-structures and their relation with occurrence of mineral matter in Ramagundam Coals, Godavari Basin, India: Implications on coal and hydrocarbon industries. J. Geol. Soc. India.

[55] Yu Y (2024). Thermal extraction of coal and derivatives to prepare hot-pressed coal briquette for COREX application. Fuel.

[56] Van Krevelen DW (1961). Coal.

[57] Moldoveanu SC, Moldoveanu SC (2021). Analytical pyrolysis of several organic geopolymers. Techniques and Instrumentation in Analytical Chemistry, Analytical Pyrolysis of Natural Organic Polymers.

[58] Durand B, Paratte M (1983). Oil potential of coals: A geochemical approach. Geol. Soc. Lond. Spec. Publ..

[59] Saxby JD (1980). Atomic H/C ratios and the generation of oil from coals and kerogens. Fuel.

[60] Petersen HI, Nytoft HP (2006). Oil generation capacity of coals as a function of coal age and aliphatic structure. Org. Geochem..

[61] Petersen HI, Suarez-Ruiz I, Filho JGM (2017). Source rock, types and petroleum generation. Geology: Current and Future Developments.

[62] Mastalerz, M., Drobniak, A., Hower, J.C. & O’Keefe J.M.K. Spontaneous Combustion and Coal Petrology. Coal and Peat Fires: A Global Perspective. Volume 1: Coal–Geology and Combustion, 2011, 47–62. 10.1016/B978-0-444-52858-2.00003-7 (2011).

[63] ECE-UN (International Classification of in-Seam Coals). Committee on Sustainable Energy; UN-ECE. Committee on Energy. Working Party on Coal; Group of Experts on the Utilization and Preparation of Solid Fuels. (1998).

[64] Stach E (1982). Stach’s Textbook of Coal Petrology.

[65] Crosdale PJ, Beamish B, Valix M (1998). Coalbed methane sorption related to coal composition. Int. J. Coal Geol..

[66] Bannerjee M (2022). Facets of coalbed methane reservoir in East Bokaro Basin, India. J. Petrol. Sci. Eng..

[67] Frazer JP (1877). Classification of coals. Am. Inst. Min. Eng..

[68] Edress NAA, Abd El-Fatah AR (2018). Fuel analyses and rank determination of the Egyptian Maghara main coal seam, north central Sinai, Egypt. Egypt. J. Petrol..

[69] Espitalie J, Deroo G, Marquis F (1985). Rock-Eval pyrolysis and its applications (Part One). Rev. Inst. Fr. Petrol..

[70] Peters KE, Peters KE (1998). Rock-eval pyrolysis. Geochemistry. Encyclopedia of Earth Science.

[71] Panwar DS, Saxena VK, Chaurasia RC, Singh AK (2017). Prospective evaluation of coal bed methane in Raniganj coal field, India. Energy Sour. A.

[72] Panwar D, Saxena V, Rani A, Singh A, Kumar V (2017). Source rock evaluation of the Gondwana coals in Raniganj Coalfield, India. Energy Sour. A.

[73] Welte D, Tissot P (1984). Petroleum Formation and Occurrence.

[74] Fowler MG, Snowdon LR, Stewart KR, McAlpine KD (1991). Rock-Eval/TOC data from five wells located within Jeanne d'Arc Basin, offshore Newfoundland. Geol. Surv. Can. Open File Rep..

[75] Li S (2022). Oil generation model of the liptinite-rich coals: Paleogene in the Xihu Sag, East China Sea Shelf Basin. J. Petrol. Sci. Eng..

[76] Singh PK (2016). Studies on thermal maturity and hydrocarbon potential of lignites of Bikaner-Nagaur basin, Rajasthan. Energy Explor. Exploit..

[77] Cudmore JF, Cudmore JF (1977). Evaluation of coals for conversion to liquid hydrocarbons. International Coal Borehole Evaluation.

[78] Singh PK (2022). Applicative coal petrology for industries: New paradigms. J. Geol. Soc. India.

[79] Tyson RV (1995). Sedimentary Organic Matter: Organic Facies and Palynofacies.

[80] Kumar S (2019). Geochemical attributes, pore structures and fractal characteristics of Barakar shale deposits of Mand-Raigarh Basin, India. Mar. Pet. Geol..

[81] Moore T (2012). Coalbed methane: A review. Int. J. Coal Geol..

[82] Peters, K.E. & Cassa, M.R. Applied source rock geochemistry. In: Magoon, L.B., Dow, W.G. (Eds), The Petroleum System-From Source to Trap. *Am. Ass. Pet. Geol. Bull,* **60,** 93–120 (1994).

[83] Wilkins RWT, George SC (2002). Coal as a source rock for oil: A review. Int. J. Coal Geol..

[84] Hunt JM (1996). Petroleum Geochemistry and Geology.

[85] Wang S, Zhang X, Lin Y, Sha Y (2019). Hydrocarbon-generated potential of bark coal components from Southern China. J. Therm. Anal. Calorim..

[86] Guo Q, Littke R, Zieger L (2018). Petrographical and geochemical characterization of sub-bituminous coals from mines in the Cesar-Ranchería Basin, Colombia. Int. J. Coal Geol..

[87] Bakr MY, Thompson GE, Burchillb P, Jonesb MA (1996). Unusually high extraction yield from the liquefaction of an Egyptian coal from Al Maghara coalfield. Fuel Process. Technol..

